# How I do it—Helsinki style mini-pterional craniotomy for clipping of middle cerebral artery bifurcation aneurysms

**DOI:** 10.1007/s00701-022-05458-6

**Published:** 2022-12-29

**Authors:** Michael Veldeman, Tobias Rossmann, Mika Niemelä, Martin Lehecka

**Affiliations:** 1 Department of Neurosurgery, University of Helsinki and Helsinki University Hospital, Topeliuksenkatu 5, 00260 Helsinki, Finland; 2 Department of Neurosurgery, RWTH Aachen University Hospital, Aachen, Germany; 3 Department of Neurosurgery, Neuromed Campus, Kepler University Hospital, Linz, Austria

**Keywords:** Intracranial aneurysm, Clipping, Middle cerebral artery bifurcation, Pterional craniotomy, Mini-pterional craniotomy

## Abstract

**Background:**

Different versions of the mini-pterional (MPT) approach have been described often with the idea the smaller the better. Attempts to reduce incision and craniotomy size for better cosmetic results should not be performed at the expense of safety.

**Method:**

We present our take on the MPT as a balance between size and safety which can be adopted by vascular neurosurgeons in training. The craniotomy stays within the confines of the superior temporal line and is completely covered by temporal muscle after closure.

**Conclusion:**

This approach is cosmetically superior while still offering anatomical familiarity and sufficient instrument maneuverability.

**Supplementary Information:**

The online version contains supplementary material available at 10.1007/s00701-022-05458-6.

## Relevant surgical anatomy

**Fig. 1:**
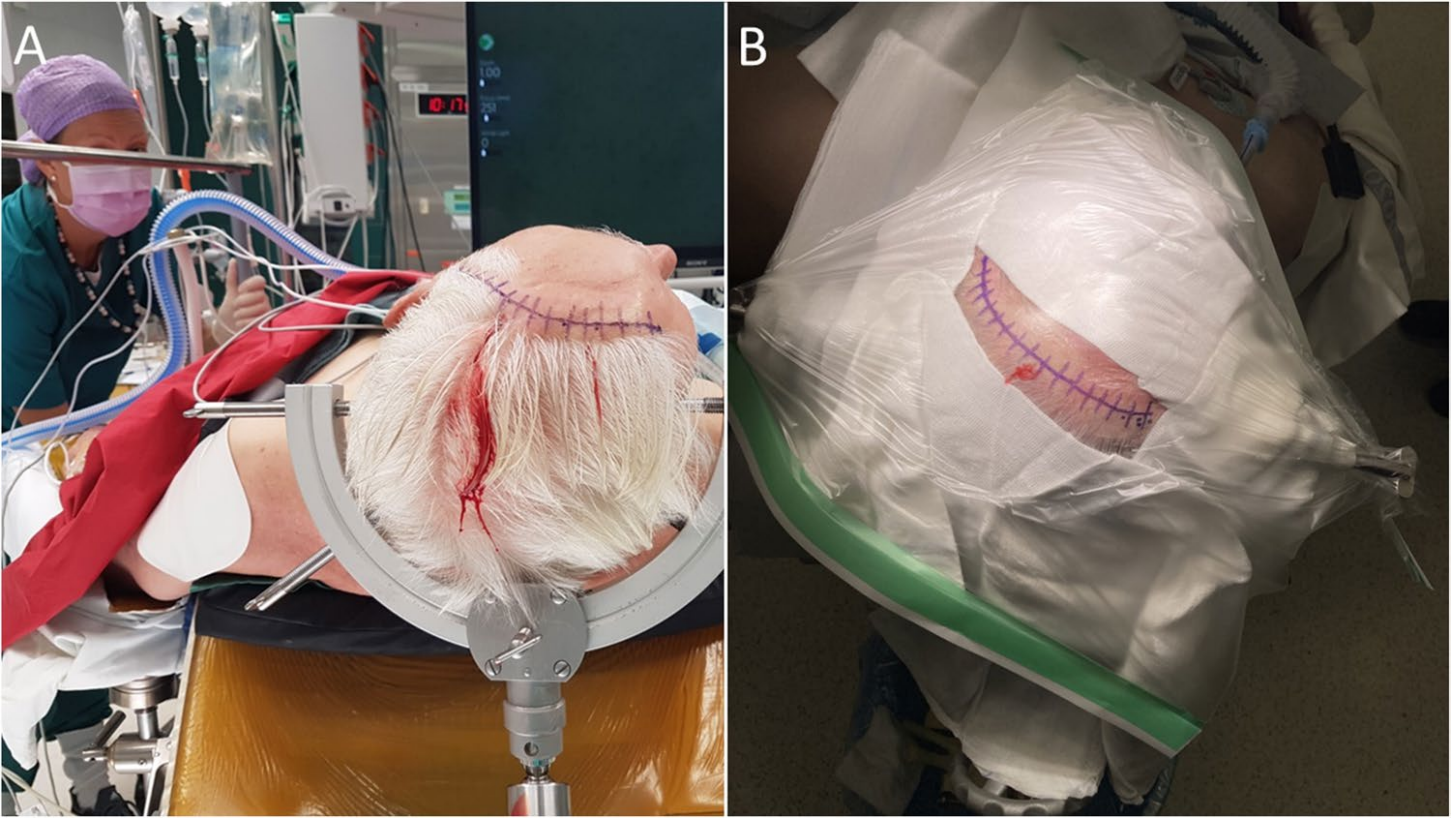
Overview of patient positioning (**A**) and planned incision (**B**) for a left sided mini-pterional approach. Note the 45-degree head rotation as appose to a 30-degree typically used for MCA bifurcation aneurysms via a standard pterional or lateral supraorbital (LSO) approach. MCA, middle cerebral artery

Incision planning is based on evident outer anatomical landmarks: zygomatic arch, external acoustic meatus, and midline. Patient positioning is illustrated in Fig. 1A. Although, linear incisions have been described [1, 4], a curved incision is preferred in our hands because it allows the incision to be completely within the haired scalp, also in people with higher or receding hairlines. The origin of the incisions lies ca. 1 cm anterior to the tragus and about 3 cm above the zygomatic arch above the frame of eyeglasses. The incision should not commence too anteriorly to prevent injuring the frontotemporal branch of the facial nerve [5, 8]. The incision then curves frontally along with and slightly behind the hairline, almost reaching the midline (Fig. 1B).

## Description of the technique

### Positioning and craniotomy

**Fig. 2:**
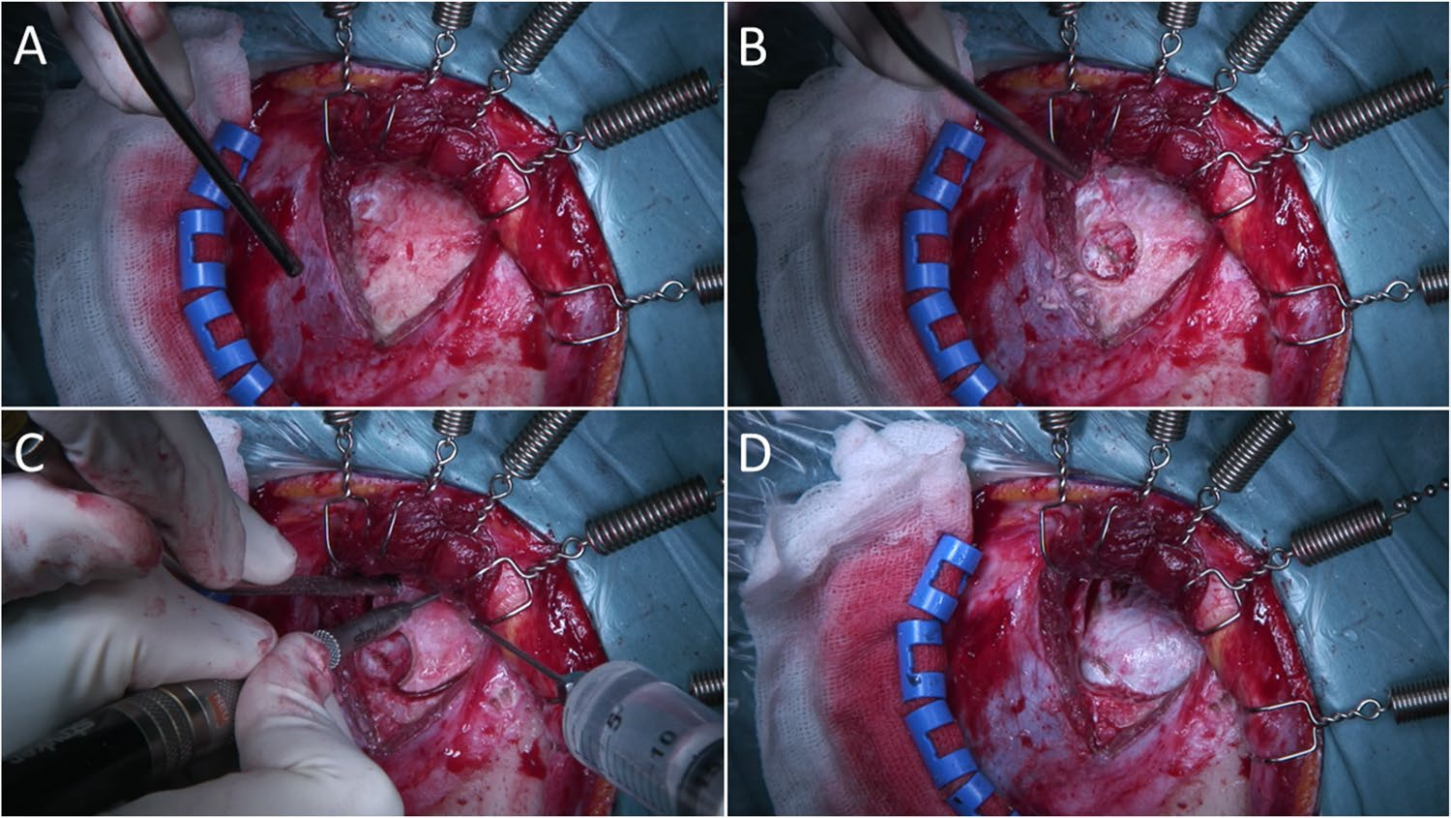
**A** The temporal muscle is incised in a V-shape by making two monopolar cuts. The muscle is dissected anteriorly and retracted. **B** One single burr hole along the posterior edge of the planned craniotomy is made. **C** The sphenoid ridge is cut by means of a craniotomy without a footplate. **D** After dural detachment, a 3-cm bone flap is removed

Skin and muscle are dissected in two layers, so the cranial insertion of the temporal muscle can be preserved. The temporal muscle is then incised in a V-shape by making two monopolar cuts (Fig. 2A). Muscle tissue is dissected anteriorly without the use of monopolar to avoid atrophy and fishhooks attached to the Sugita frame retract skin and muscle. A single burr hole is placed, along the posterior edge of the planned craniotomy (Fig. 2B). After dural detachment, a 3-cm craniotomy is completed. The craniotomy is centered over the Sylvian fissure, of which the location can be estimated based on the indentation of the temporal bone. The bone of the sphenoid wing is cut using a footplateless craniotome (Fig. 2C). The craniotome’s blade is driven anteriorly and caudally precluding the need of further extensive sphenoid drilling. Minimal drilling is performed to flatten the anterior skull base. The dura is opened toward the sphenoid and everting dural tenting sutures are placed to maximize space and minimize epidural venous oozing.

### Sylvian fissure dissection

After introduction of the microscope or exoscope, the sphenoid ridge is followed subfrontally toward the midline and the optic and carotid cisterns are identified and opened for cerebrospinal fluid (CSF) evacuation. In unruptured aneurysms, if there is sufficient space, this step can be omitted, and CSF can be drained directly from the Sylvian cistern. The Sylvian fissure is always dissected in a distal to proximal manner, regardless of the aneurysm’s rupture status. Superficial Sylvian veins are left attached to the temporal lobe, to preserve drainage into the sphenoparietal sinus or its anatomical equivalents [9]. The size of the Sylvian split is tailored but usually does not exceed 1.5 cm in unruptured middle cerebral artery (MCA) aneurysms. The arachnoid is cut on the frontal side of the superficial Sylvian vein using the blade of a hypodermic needle. Hydrodissection helps to spread the fissure prior to further dissection. The dissection is carried deeper by means of bipolar spring action and arachnoid strands are cut with microscissors [7]. The frontal M2 trunk is usually the first/easiest to identify which can then be followed until identification of the bifurcation and the aneurysm.

### Aneurysm dissection and clip positioning

**Fig. 3:**
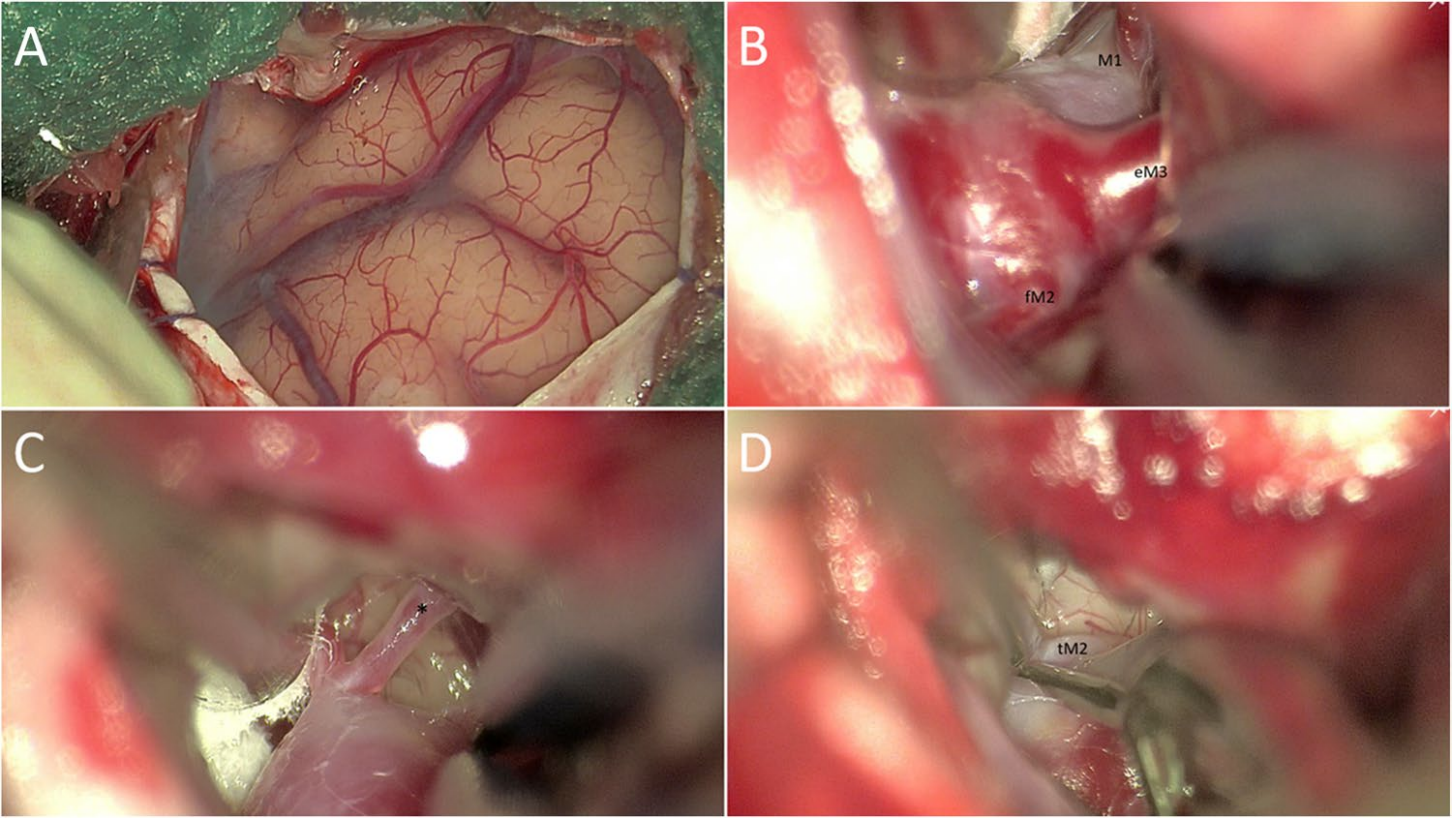
**A** Dural edges are circumferentially retracted with everting temporary sutures to optimize space. **B** The middle cerebral artery trunk (M1), frontal M2 (fM2), and the early M3 (eM3) seen as in angiographical reconstruction (Fig. 4B). **C** A small distal perforator (*), not visible on reconstructed angiographic images, requires to be dissected of the aneurysm dome. **D** Careful clip placement is carried out to avoid stenosing of the MCA bifurcation and temporal M2. eM3, early opercular segment branch; fM2, frontal insular branch of the middle cerebral artery; MCA, middle cerebral artery; M1, main trunk of the middle cerebral artery

The base of the aneurysm is dissected first, and dissection is continued proximally until the M1 is sufficiently exposed for potential temporary clipping (Fig. 3B). Once proximal control is available, the dome is dissected and perforators adherent to the dome’s surface are dissected off by means of a blunt narrow microdissector (e.g., Aesculap CASPAR FF310R, Braun SE, Melsungen, Germany) (Fig. 3C). Depending on aneurysm size, projection, and morphology, temporary M1 clipping is used to decrease dome turgor and facilitate permanent clip applications. Prior to temporary clipping, the appropriate clip is selected and introduced into the field to assess its suitability. The definitive clip is placed under careful consideration of patency of the bifurcation and especially the temporal M2 (Fig. 3D). Tandem clip maneuvers by stacking two similar clips parallel to each other allow safe adjustments without aneurysm refilling. Near-infrared indocyanine green videoangiography is used to confirm aneurysm occlusion and patency of both M2 branches [3]. The aneurysm dome is resected for research purposes whenever possible. Coagulation on and around the aneurysms dome and neck is avoided not to weaken the reconstructed vessel wall consisting of newly apposed aneurysm neck.

## Indications

The MPT approach is a safe and effective, less invasive alternative to the standard pterional craniotomy to address aneurysms of the MCA bifurcation. Though smaller (< 3 cm) approaches have been described, we believe the technique, as described above (and in the accompanying video), offers a safe balance between size and instrument maneuverability. This makes it suitable to be adopted by training vascular neurosurgeons who wish to start using smaller craniotomies for clipping procedures. We apply this type of approach for all configurations of MCA bifurcation aneurysms.

## Limitations

A smaller craniotomy means less instrument maneuverability. Appropriate head rotation is essential to prevent conflict with the craniotomy edges. Rotation of the operating table can help correct for insufficient head rotation during surgery. In case of intraoperative aneurysm rupture, the surgical corridor is narrower which makes introduction of a second suction device more difficult. However, a well-placed single suction device usually suffices to clear the field; it leaves the surgeon single-handed. Nonetheless, this approach is routinely used in our hands for ruptured aneurysms as well.

## How to avoid complications

When a frontal or temporal corticotomy is planned to evacuate an associated intracerebral hemorrhage, the craniotomy requires to be tailored accordingly. To address aneurysms of the anterior communicating artery and surrounding complex, the craniotomy should be adjusted and slightly extended anteriorly and cranially to allow a more anterior subfrontal trajectory. The approach is used in a similar fashion for M1 segment aneurysms as well, but not for more proximal (carotid terminus) aneurysm locations.

## Specific perioperative considerations

**Fig. 4:**
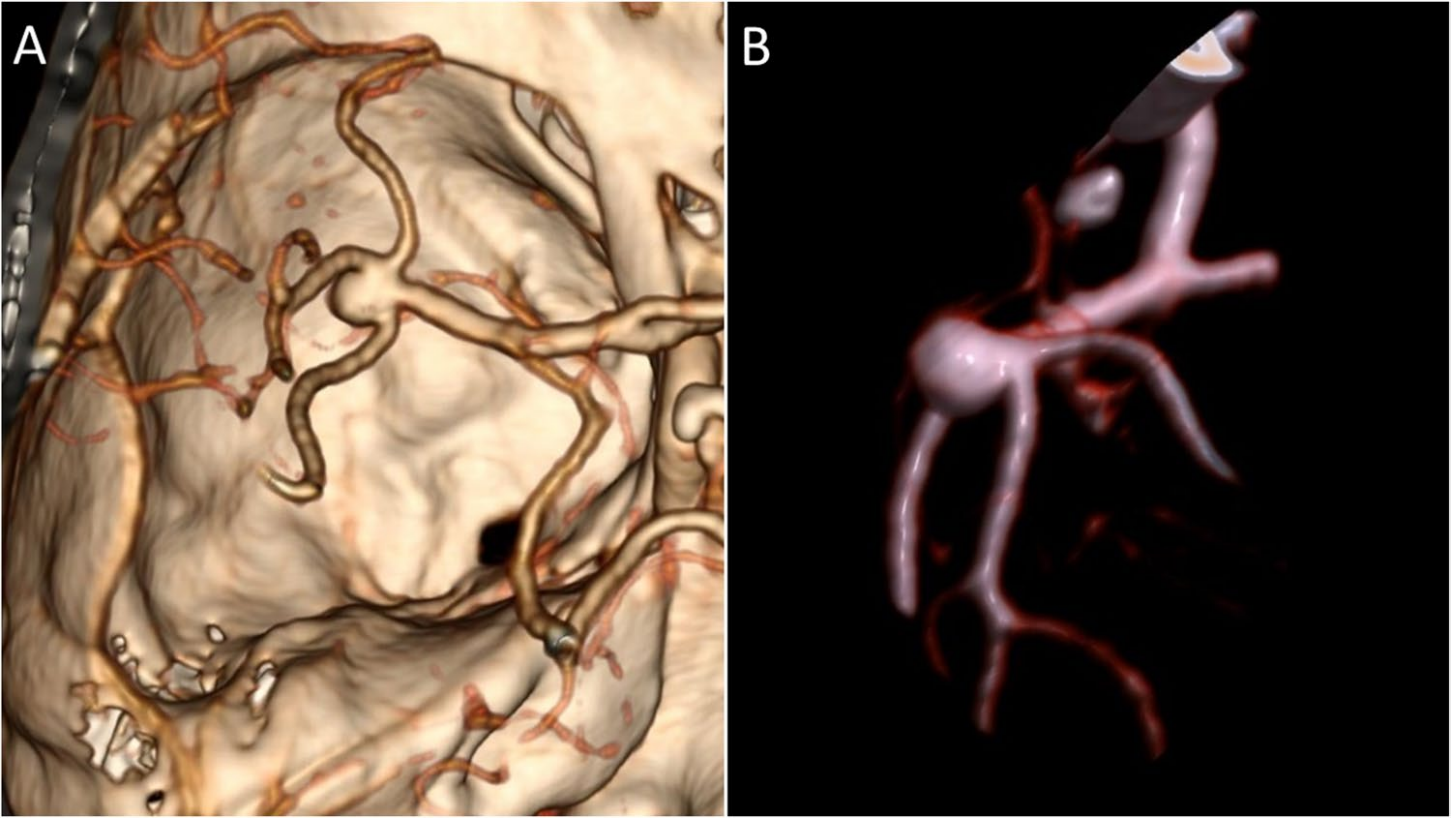
Middle cerebral artery bifurcation aneurysm with a lateral and posterior directed projection. **A** Three-dimensional reconstruction of CT angiographical images demonstrating the relationship of the aneurysm and its parent vessel, with surrounding bony landmarks. **B** Surgeon’s view of the aneurysm, M1-segmeent, and both M2 branches. An early frontal M3 branch can be appreciated. CT, computed tomography; M1, main trunk of the middle cerebral artery; M2, insular branch of the middle cerebral artery; M3, opercular segment of the middle cerebral artery

We recommend careful study of angiographic imaging with three-dimensional reconstruction and rotation of the model as it will be encountered during surgery (Fig. 4A, B). Major points of attention are the length and orientation of the M1 segment and resulting position of the MCA bifurcation in relation to the limen insulae. Short M1 segments require more pronounced head rotation of up to 60 degrees to allow proper exposure, whereas for longer segments, 45 degrees rotation is sufficient. This is as appose to the lateral supraorbital approach (LSO) for MCA bifurcation aneurysms, for which less head rotation is required [6, 10]. Familiarization with the individual relationship between the aneurysm’s projection and sphenoid wing can be of great help to tailor the necessary bony exposure. Upward or downward bending of the M1 oftentimes results in frontal or temporal projection of the aneurysm’s dome, respectively, and possible dome adhesion to the corresponding lobe [2].

## Specific information to give to the patient about surgery and potential risks

General discomfort, headache, and mastication problems are most probably milder compared to a standard pterional approach. Alternative minimal-invasive approaches such as the supraorbital eyebrow incision can be discussed with the patient, but the decision should be guided by individual anatomy (dome projection and extension of paranasal frontal sinuses) and surgeon’s experience and familiarity as well as anticipated cosmetic result based on eyebrow size and density.

## Supplementary Information

Below is the link to the electronic supplementary material.

Supplementary file1 The accompanying video material describes technical pearls and pitfalls of the “Helsinki Style”-mini-pterional approach, in an illustrative case. (MP4 44319 KB)

## Abbreviations

CSF: Cerebrospinal fluid
LSO: Lateral supraorbital
M1: Horizontal segment of the middle cerebral artery
M2: Insular segment of the middle cerebral artery
M3: Opercular segment of the middle cerebral artery
MCA: Middle cerebral artery
MPT: Mini-pterional approach

## References

[1] Cabrilo I, Schaller K, Bijlenga P (2015). How mini can minipterional craniotomies get?. Neurosurgery.

[2] Dashti R, Hernesniemi J, Niemelä M, Rinne J, Porras M, Lehecka M, Shen H, Albayrak BS, Lehto H, Koroknay-Pál P, de Oliveira RS, Perra G, Ronkainen A, Koivisto T, Jääskeläinen JE (2007). Microneurosurgical management of middle cerebral artery bifurcation aneurysms. Surg Neurol.

[3] Dashti R, Laakso A, Niemelä M, Porras M, Hernesniemi J (2009). Microscope-integrated near-infrared indocyanine green videoangiography during surgery of intracranial aneurysms: the Helsinki experience. Surg Neurol.

[4] Esposito G, Dias SF, Burkhardt JK, Fierstra J, Serra C, Bozinov O, Regli L (2019). Selection strategy for optimal keyhole approaches for middle cerebral artery aneurysms: lateral supraorbital versus minipterional craniotomy. World Neurosurg.

[5] Krayenbühl N, Isolan GR, Hafez A, Yaşargil MG (2007). The relationship of the fronto-temporal branches of the facial nerve to the fascias of the temporal region: a literature review applied to practical anatomical dissection. Neurosurg Rev.

[6] Lehecka M, Laakso A, Hernesniemi J (2011) Microneurosurgery basics and tricks, 1st edn. Helsinki

[7] Muhammad S, Tanikawa R, Lawton M, Regli L, Niemelä M, Korja M (2019). Microsurgical dissection of Sylvian fissure-short technical videos of third generation cerebrovascular neurosurgeons. Acta Neurochir.

[8] Rodriguez Rubio R, Chae R, Vigo V, Abla AA, McDermott M (2019). Immersive surgical anatomy of the pterional approach. Cureus.

[9] San Millán Ruíz D, Fasel JH, Rüfenacht DA, Gailloud P (2004). The sphenoparietal sinus of Breschet: does it exist? An anatomic study. AJNR Am J Neuroradiol.

[10] Veldeman M, Rossmann T, Korja M, Laakso A, Lehecka M, Niemelä M (2022). A new home for the Helsinki Neurosurgical Department - closure of Töölö Hospital after 90 years of neurosurgical history. Acta Neurochir.

